# The clinical research of 5 steps sequential method for whole treatment of hemorrhagic radiation cystitis in china

**DOI:** 10.7150/ijms.47906

**Published:** 2021-01-01

**Authors:** Zhenghua Ju, Wenchang Yu, Youyuan Li, Weiqing Han, Xinhua Tu, Shaoxing Zhu, Qing Zou

**Affiliations:** 1 Department of Urology, Fujian Medical University, Fujian Cancer Hospital, Fuzhou, China.; 2 Department of Invasive Technology, Fujian Medical University, Fujian Cancer Hospital, Fuzhou, China.; 3 Department of Urology, Hubei Provincial Cancer Hospital, Wuhan, China.; 4 Department of Urology, Hunan Provincial Cancer Hospital, Changsha, China.; 5 Department of Urology, Jiangxi Provincial Cancer Hospital, Nanchang, China.; 6 Department of Urology, Zhejiang Provincial Cancer Hospital, Hangzhou, China.; 7 Department of Urology, Jiangsu Provincial Cancer Hospital, Nanjing, China.

**Keywords:** Hemorrhagic cystitis, Sodium hyaluronate, transurethral electrocoagulation, transcatheter arterial embolization, hyperbaric oxygen

## Abstract

**Background:** Curing hemorrhagic cystitis remains a challenge. We explore a continuous and effective treatment for hemorrhagic radiation cystitis.

**Methods:** The data of patients in 6 provincial cancer hospital urology departments between April 2015 and December 2019 was reviewed retrospectively. Patients were classified as moderate and severe groups. The 5-steps sequential method was adopted. Two groups were initiated with step 1 and step 3 respectively. Step 1 was symptomatic treatment. Thrombin solution or sodium hyaluronate was administrated for bladder irrigation in step 2. Step 3 was transurethral electrocoagulation. Step 4 was interventional embolization. Step 5 was HBO therapy. OABSS was used to assess the improvement of patients' symptoms. The outcome was evaluated after at least 6 months of follow-up.

**Results:** A total of 650 patients (56 men and 594 women), mean age 71.2 years, were enrolled in the 5 steps sequential method. 582 patients were classified as moderate and 68 severe group. In moderate group, the cure rate of step 1 was 61.2% (356/582), 80.4% (468/582) after step 2, 93.1% (542/582) after step 3, 96.2% (560/582) after step 4, and 99.8% (581/582) after step 5. In severe group, the cure rate was 54.4% (37/68) after step 3, 76.5% (52/68) after step 4, and 94.1% (64/68) after the step 5 respectively. The mean OABSS scores of both groups significantly decreased after 5 steps sequential method treatment (P<0.01).

**Conclusions:** Our results show hemorrhagic radiation cystitis can be cured in 5 steps, and the 5 steps sequential method is welcomed and effective. Therapy efficacy depends on the number of steps adopted and the severity of hematuria.

## Introduction

Radiation cystitis is a late complication of radiotherapy for pelvic malignancies and occurs at least 3 months after the initiation of radiation treatment but may occur in a delayed manner even beyond 10 years.^1^ Treatment of hemorrhagic cystitis remains a challenge for urologists. 2 The reason is that various symptoms lead to different treatments, such as bladder irrigation, transurethral electrocoagulation, transcatheter arterial embolization, and hyperbaric oxygen (HBO) therapy and so on. However, there is no undoubted clinical evidence with any guarantee of long-term efficacy. 3 Furthermore, there are no firm guidelines to follow. 4 From April 2015 to December 2019, six provincial cancer hospital urology departments in China were motivated to explore the effectiveness of the five-step sequential method with the assistance of the Urologic Chinese Oncology Group (UCOG).^5^ Total 650 patients were involved in our treatment, with 162 cases in Fujian province cancer hospital, 135 cases in Hubei province, 101 cases in Hunan province, 100 cases in Jiangxi province, 77 cases in Zhejiang province, 75 cases in Jiangsu province respectively. All patients experienced a complete or partial resolution of hematuria after our 5-steps sequential method. We think our method is worthy of reference and promotion in treating hemorrhagic cystitis.

## Materials and Methods

This study was approved by the ethics board of the Fujian Medical University, Fujian Cancer Hospital (KT-2014-004-11), and all patients provided written informed consent. Patient information was anonymised and de-identified prior to the study, and we carried out all study procedures in accordance with the Declaration of Helsinki guidelines. All methods in this study were performed in accordance with the relevant guidelines and regulations.

**Figure 1:**
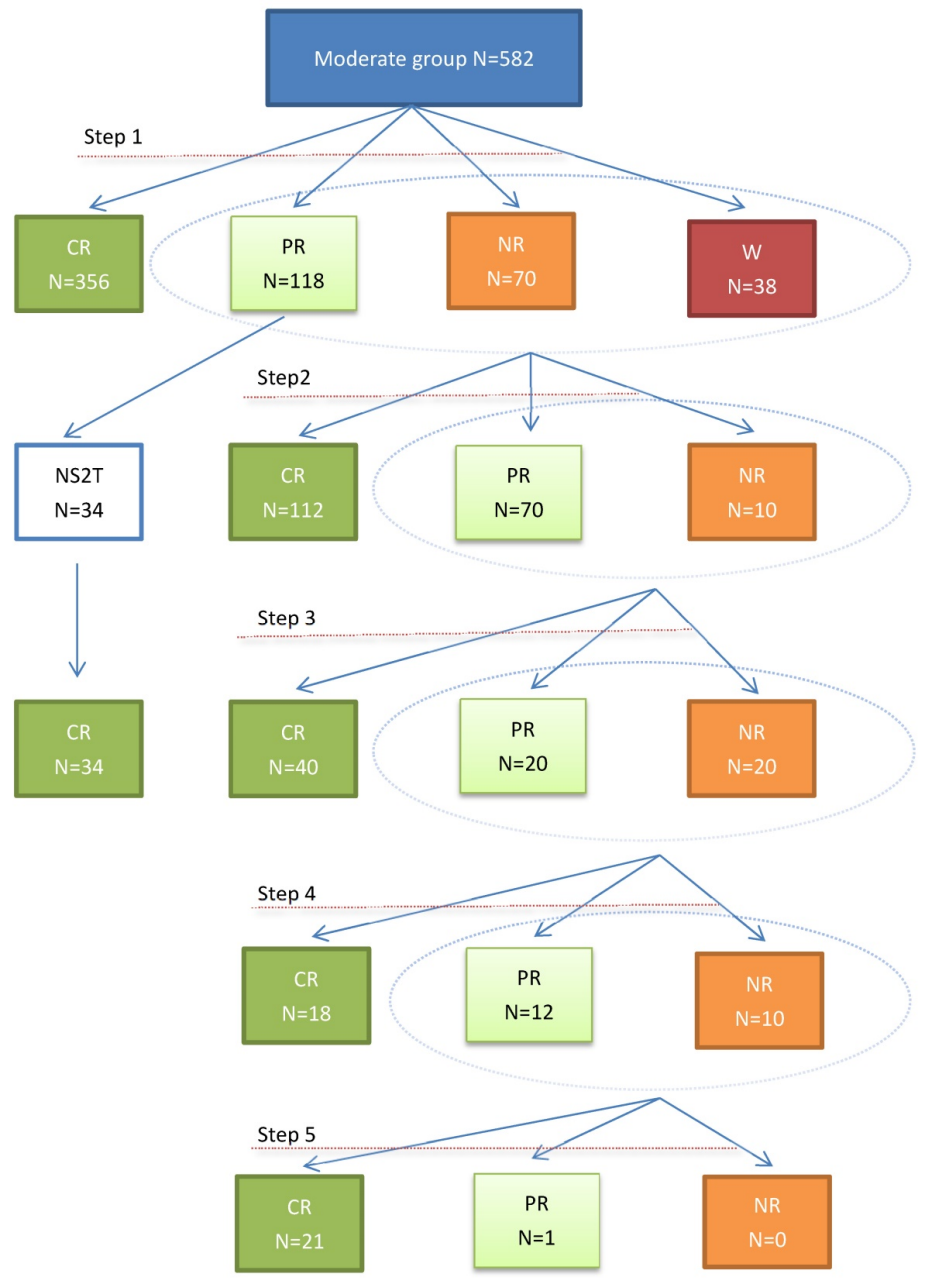
** Treatment process in moderate group.** no step 2 treatment: NS2T; complete resolution: CR; partial resolution: PR; no resolution: NR; worsening: W

The data of 650 patients were retrospectively reviewed. Before 5-steps sequential method, a thorough evaluation would be done to determine the cause. If an etiology was not obvious, the patient should undergo hematuria workup including urine cytology, upper tract imaging and cystoscopy. The patient's current medications should be reviewed and anticoagulants would be stopped. Patients with thrombocytopenia and other coagulation abnormalities should be corrected. Laboratory evaluation included hemoglobin, complete blood count, blood urea, serum creatinine, and coagulation profile and urine culture. Hemorrhagic patients with Radiation Therapy Oncology Group (RTOG) and European Organization for Research and Treatment of Cancer (EORTC) grade 1-3 and grade 4-5 were stratified into moderate and severe groups respectively 6 and the moderate treatment process is shown in Figure 1.

**Step 1:** Symptomatic treatment. Tolterodine and analgesics were helpful in alleviating symptoms, and PAMBA, E-aminocaproic acid or other hemostatic agents was for hemorrhage. Antibiotics were used to prevent infection. Anemia patients with hemoglobin less than 90g/L were appropriately given component blood transfusion to maintain hemodynamical stability. If complete or partial resolution standards were reached after one month treatment, the patient would be discharged after consulting the patient's opinion. If no change or worsening hematuria occurred, the patient would be transferred to next step.

**Step 2:** Bladder instillation treatment: thrombin solution (thrombin 1000U + sodium chloride injection 40ml) was perfused into the bladder once a day for 5 consecutive days. Or 40mg of sterile sodium hyaluronate was used and kept for 60min once a week, a total of 4 times. The treatment time was limited to one month.

**Step 3:** Transurethral electrocoagulation. After step 2 treatment, patients in moderate group without obvious remission were transferred to step 3. Severe cases were transferred to step 3 directly, symptomatic treatments were prescribed if necessary. The bladder mucosa hemorrhage was coagulated and Bladder bleed clot was removed simultaneously. If the remission was not obvious after 1 week, the patient will be transferred to step 4.

**Step 4:** Transcatheter arterial embolization. After consultation in the interventional department, selective bilateral internal iliac artery intra-pelvic branch embolization was performed.

**Step 5:** HBO therapy. Patients received HBO therapy with 90 minutes of 100% oxygen breathing at 2.36 atm absolute pressure per session, including 5-minute air breaks after every 30 minutes of oxygen. An initial course of 40 treatments was planned, one session daily, five times weekly.

The clinical outcome measures after 5 steps therapy included symptomatic assessment (complete resolution, partial resolution, no resolution, and worsening hematuria) by physicians or as reported by the patients with the way of follow-up forms. Overactive Bladder Symptom Scores (OABSS) was used to evaluate the bladder function of patients before and after treatment according to the report of Homma et al.^7^ Clinical improvement was defined as complete or partial resolution of macroscopic hematuria. Complete resolution referred to the absence of macroscopic hematuria, clear urine color, no macroscopic hematuria, urine routine erythrocyte 0 - (+) /HP, cystoscopy showed that the bladder mucosa was basically intact, no bleeding spots and ulcerative necrotic surface. Partial resolution referred to a reduction in the severity or frequency of macroscopic hematuria that corresponded to a change to a lower grade of radiotherapy-related hematuria. The degree of hematuria and the number of routine erythrocytes in urine decreased, and cystoscopy showed that the bladder mucosa hyperemia points and the ulcer decreased too. Total resolution rate = complete resolution rate + partial resolution rate. The follow-up included routine urinalysis and cystoscopy theoretically. Within half a year after the completion of the treatment, the urine routine was reviewed once a month, and once every three months after that. Cystoscopy is performed every three months, a total of four times. If necessary, review pelvic CT to understand the pelvic situation. Microscopic examination of urine was not evaluated punctually because some patients were followed up by postal outcomes surveys without returning to the clinician's office actually. The results of the post-treatment cystoscopic evaluation were not evaluated regularly because most patients with symptomatic improvement would not undergo repeat cystoscopy because of the suffering. We generally consider no bleeding for six months and no abnormalities in cystoscopy and routine urine tests to be considered free of recurrence.

The data were analyzed using Statistical Package for Social Sciences, version 11.0, with the one-sided Fisher's exact test in two by two tables and the two-sided Pearson chi-square test with two degrees of freedom in the three by two table. Statistical significance was reached at a P value of 0.05 or less.

## Results

A total of 650 patients (56 men and 594 women), mean age 71.2 years, were enrolled, among them, 43 had prostate cancer, 13 had bladder cancer, and 594 had cervical cancer. The average dose of radiotherapy is 60Gy (range 20 to 120). Mean time between completion of radiation therapy and onset of hematuria was 46.7 months (range0.5 to 480). All patients finished the treatment. 582 patients and 68 patients were divided into moderate and severe groups respectively. In moderate group, after step 1, 356 in 582 patients with evaluable data experienced complete resolution and marked improvement of hematuria, 118 got partial Resolution, 70 got no change, 38 got worsening respectively, and total resolution rate was 61.2%. The patients with no change or worsening and 84 in 118 got partial resolution who had higher expectations were turned to step 2, and 34 patients in 118 got partial resolution entered the third step of treatment without the second step. As the treatment progressed, 581 patients got complete and 1patient got partial resolution after Step 5 (graph 1 and table 1).

The OABSS of patients receiving step 1 in moderate group decreased from 7.7±1.1 to 3.2±1.2 (*P* <0.01). The patient with higher score before sequential treatment meaning more steps had to be accepted, and the score was from 8.4±1.1, to 8.9±1.2, 9.9±2.0, 11.7±2.1, 12.9±1.9 respectively (table 2). No complications were observed in moderate group.

In severe group, starting from step 3, 37 in 68 patients got complete resolution, 25 got partial resolution, 6 got no change, 0 got worsening respectively, and resolution rate was 54.4%. 6 patients with no resolution and other 24 in 25 got partial resolutions with high expectation were turned to step 4, one patient with partial resolution died of tumor recurrence, and cumulative resolution rate was 76.5%. 94.1% patients got complete resolution after the ending of Step 5 (table 3).

The OABSS scores of severe group decreased from 13.9±2.1 to 5.3±1.3 after receiving step 3 (table 4). Higher score before sequential treatment meaning more steps had to be accepted as in moderate group. 16 patients who previously had partial Resolution underwent treatment of step 1 for recurrent symptoms. No major complications were observed in 16 patients. All the 68 patients in severe group underwent post-sequential treatment cystoscopic evaluation to visually assess response in 1 month.

## Discussion

Severe hemorrhagic radiation cystitis is an uncommon, but potentially devastating, side effect of pelvic radiotherapy. Tissues undergo a progressive deterioration influenced by a reduction of small blood vessels and increased fibrosis, replacing the normal tissue until localized hypoxia compromises normal function.^8^ Subsequently, diffuse edema, telangiectasia, and interstitial fibrosis leads to further symptoms of varying severity.^9^

Most patients with bladder hyperemia in China used to prefer to cancer hospital urology department because they adopted their radiation therapy in the same hospital. In fact, when we started treating bladder bleeding, we had no experience and had to copy internationally established treatment protocols. For example, hyperbaric oxygen therapy, we've found that it's expensive, and a lot of patients don't want to accept it. Or, in severe cases, conservative drug therapy is often less effective. Many patients seek treatment in China's top hospitals, but many still do not receive effective treatment. Therefore, from April 2015 to November 2019, 6 provincial cancer hospital urology departments with radiation cystitis hemorrhage patients were motivated to explore the efficacy of 5 steps sequential method. The first is oral medication, which is easily accepted by patients and costs little. Then perfusion, which is relatively painless. The electrocoagulation, which requires hospitalization. Interventional therapy requires the treatment of other departments. There are no relevant departments of hyperbaric oxygen therapy in Chinese cancer hospitals generally, which requires treatment in general hospitals and costs a lot of money, so we arrange it at the end. Since 2018, after we introduced our methods to our colleagues in China, so far more than 20 provincial cancer hospitals in China have accepted our methods in urology. It can be said that in China, the past disordered treatment has been basically changed. But we still have a lot of problems at early stage. The first problem we faced was how we stratify patients according to the severity of their symptoms. The RTOG and EORTC scoring criteria was commonly used in clinical trials to describe acute or late radiotherapy- related morbidities, however, according to our observation, this standard was too strict to be applied in clinical practice, and dividing patients into five groups is also inconvenient for experimental studies. Therefore, the patients were divided into two groups conveniently. Furthermore, in our research, the vast majorities of cases are mild and can be cured by outpatient treatment in step 1. Expensive treatments such as hyperbaric oxygen are often unnecessary. Only severe cases require multi-step treatment. Patients with moderate hemorrhage predictably got complete resolution or partial resolution after receiving symptomatic treatment and bladder irrigation therapy while the correct understanding of the disease was given. On the contrary, patients with severe symptoms showed an uncooperative attitude for the slow treatment effect of steps1, 2. As result, we take a different starting point for the whole treatment.

Another bright spot is that unlike in the past when there was no definitive treatment, we told patients about our step-by-step strategy of sequential methods at the early stage of treatment, resulting better understanding of the process and higher obedience. In fact, the curative effect of symptomatic treatment was limited in severe group even for a long time. However, most patients showed great willing to 1 or 2 steps of treatment and only after the effect was not so good they had to accept the next step.

Irrigation therapy is simple to operate and convenient for community doctors, and the short-term effect is better than that of symptomatic treatment. In the past, alum or formaldehyde solution was infused into the bladder, which had great side effects and a high rate of re-bleeding. Although these drugs were easy to obtain in china, we gradually abandoned them for the suffering. In recent years, sodium hyaluronate in bladder irrigation has become increasingly popular. The small molecule of hyaluronic acid can infiltrate dermis, can dilate blood capillary, increase haemal circulation, and promote epidermal cell proliferation at the same time. Currently, it is widely used in bladder tumor, trauma, urinary retention and cystitis. 10 However, the high price of sodium hyaluronate limited further promotion. In the past 3 years, only 34 patients received sodium hyaluronate for instillation. Thrombin injection had a certain hemostatic effect, which could promote blood coagulation to achieve the purpose of local hemostasis, it has a limited therapeutic effect. In patients with poor economic conditions, it is also a good choice.

If step 2 is not effective, indicating there may be active arteriorrhagia, electrocoagulation should be performed in time. Electrocoagulation can be performed under local anesthesia or epidural anesthesia, and bipolar plasma system is recommended. In bipolar plasma system, the heat penetration depth is relatively shallow during operation, and the operation can be repeated. In severe group, a patient was treated with electrocoagulation for 3 times. Zhu et al 11 reported their experience in 10 patients and suggest that transurethral coagulation using Greenlight potassium-titanyl-phosphate laser is a well-tolerated and effective strategy for the treatment of hemorrhagic radiation cystitis with the power setting limited to 20-30W. In our study, the majority of moderate patients can be cured by the first 3 steps.

If the remission was not obvious after the first 3 steps, it would be transferred to step 4. We used bilateral iliac artery branch embolization, although the short-term effect was acceptable, however, a wide range of collateral circulation formed gradually, and which would lead to hematuria relapse. Relevant literature reported, the hematuria relapse rate was up to 50% of patients.

Numerous studies have reported the use of HBO in patients with hemorrhagic radiation cystitis, with varying results, length of follow up and treatment protocols. HBO therapy has been extensively investigated in the management of radiation induced injuries. HBO is helpful for the formation of new blood vessels in the bladder, improving tissue oxygen supply, and reversing the pathological process of radioactive cystitis. Urethral bleeding and pain could be effectively controlled, and 70% of hemorrhagic radioactive cystitis patients could be cured.^12^-^17^ However, the main reason HBO therapy was be listed as the last place was the symptoms of patients with radioactive cystitis and hemorrhage were urgent, while the effect of sole HBO therapy was slow. After the treatment of the first 4 steps, even if the complete resolution were not achieved, there was partial resolution definitely. Hence, patients would receive 40 hyperbaric oxygen sessions with patience. Another drawback at present of HBO therapy was probably the high cost of treatment even in china.

While most studies used symptomatic hematuria as an outcome measure, some reports described cystoscopic findings before or after treatment. We believed this is not enough and not exact, OABSS was adopted in our study because it was easy to quantify the improvement in symptoms.

The limitations of this study were mainly retrospective studies, but good results were achieved. In China, because bladder bleeding does not receive the same attention as tumor diseases, patients tend to control symptoms and finish treatment as soon as possible. In addition, our hierarchical diagnosis and treatment is not perfect. So if at the beginning of the treatment, the treatment has not achieved significant results, it is difficult for doctors to gain the trust of patients. Patients are more likely to choose a more authoritative hospital for further treatment blindly. As a result, doctors will use the most effective way they think to control symptoms, and it was difficult to enroll control patients. Plus, our doctors found it difficult to get in touch with patients once symptoms improve, follow-up was unusually difficult, and Randomized controlled studies are harder to do. In the future work, it may be the focus of our work to select suitable patients for prospective study. At that time, our data will be more convincing. After nearly 3 years of clinical practice, the 5-step sequential treatment of hemorrhagic radiation cystitis conformed to the law of disease development and treatment ideas, and was easily accepted by patients. From the view of patients, individualized and humanized treatment might be more acceptable and effective.

## Author Contributions

Zhenghua Ju: Study conception and design, conducting experiment and acquiring data, analyzing and interpreting data, drafting manuscript, and critical revision.

Wenchang Yu: Conducting experiment and acquiring data.

Youyuan Li: Conducting experiment and acquiring data.

Weiqing Han: Conducting experiment and acquiring data.

Xinhua Tu: Conducting experiment and acquiring data.

Shaoxing Zhu: Conducting experiment and acquiring data.

Qing Zou: Conducting experiment and acquiring data.

## Figures and Tables

**Table 1:** Therapeutic effect in moderate group

	N	Complete resolution	Partial resolution	No change	worsening	Cumulative complete cure rate
After Step1	582	356	118	70	38	(356/582)61.2%
After Step 1+2	192	112	70	10	0	(468/582)80.4%
After Step1+3 and Step 1+2+3	114	74	20	20	0	(542/582)93.1%
After Step 1+2+3+4	40	18	12	10	0	(560/582)96.2%
After Step 1+2+3+4+5	22	21	1	0	0	(581/582)99.8 %

**Table 2:** OABSS in moderate group

	Step 1	Step 1+2	Step 1+3	Step 1+2+3	Step 1+2+3+4	Step 1+2+3+4+5
before treatment	7.7±1.1	8.4±1.1	8.9±1.2	9.9±2.0	11.7±2.1	12.9±1.9
after treatment	3.2±1.2	3.4±0.7	3.7±0.8	4.2±1.4	5.3±1.9	5.1±1.9
*P*	*P<*0.01	*P<*0.01	*P<0.01*	*P<*0.01	*P<*0.01	*P<*0.01

**Table 3:** Therapeutic effect in severe group

	N	Complete resolution	Partial resolution	No change	worsening	Cumulative complete cure rate
Step 3	68	37	25	6	0	54.4%
Step 3+4	30	15	8	7	0	76.5%
Step 3+4+5	15	12	3	0	0	94.1%

**Table 4:** Change of OABSS in severe group

	Step 3	Step 3+4	Step 3+4+5
before treatment	13.9±2.1	14.7±2.5	15.1±2.4
after treatment	5.3±1.3	5.9±1.7	6.2±1.7
*P*	*P<*0.01	*P<*0.01	*P<*0.01

## Abbreviations

HBO: Hyperbaric oxygen
OABSS: Overactive Bladder Symptom Scores
